# Gonad RNA-specific qRT-PCR analyses identify genes with potential functions in schistosome reproduction such as SmFz1 and SmFGFRs

**DOI:** 10.3389/fgene.2014.00170

**Published:** 2014-06-10

**Authors:** Steffen Hahnel, Thomas Quack, Sophia J. Parker-Manuel, Zhigang Lu, Mathieu Vanderstraete, Marion Morel, Colette Dissous, Katia Cailliau, Christoph G. Grevelding

**Affiliations:** ^1^Biologisch-Medizinisches Forschungszentrum Seltersberg, Institute of Parasitology, Justus-Liebig-UniversityGiessen, Germany; ^2^CIIL - Center of Infection and Immunity of Lille, CNRS-UMR 8204, INSERM U1019, Institut Pasteur de Lille, Université Lille Nord de FranceLille Cedex, France; ^3^Laboratoire de Régulation des Signaux de Division, EA 4479, IFR 147, Université Lille 1 Sciences et Technology, Villeneuve d'Ascq CedexFrance

**Keywords:** schistosomiasis, *Schistosoma mansoni*, development, helminth reproduction, gonad, frizzled (Fz), fibroblast growth factor receptor (FGFR), organ isolation

## Abstract

In the search for new strategies to fight schistosomiasis, the unique reproductive biology of *Schistosoma mansoni* has come into the focus of research. The development of the gonads and the ability of egg production are fundamental not only for continuing the life cycle but also for pathogenicity. Previous studies of schistosome biology demonstrated an influence of pairing on gonad development of the female and on gene expression profiles in both genders. Due to the limited access to specific tissues, however, most of these studies were done at the level of whole worms neglecting individual tissues that may be targets of pairing-dependent processes. Recently, we established a protocol allowing the isolation of testes and ovaries from adult *S. mansoni*. Here, we describe tissue-specific qRT-PCR analyses comparing transcript levels of selected genes on the basis of RNA from gonads and whole worms. Gene expression in ovary and testes was in some cases found to be significantly influenced by pairing, which was not traceable in whole worms. Among the candidate genes identified as regulated by pairing in gonads were the frizzled homolog SmFz1 and the two fibroblast growth factor receptor homologs SmFGFR-A and SmFGFR-B. First functional characterizations were done, including comparative qRT-PCR analyses, *in situ*-localization experiments, heterologous expression in *Xenopus* oocytes (SmFGFR-A/B), and inhibitor studies using the Fz/Dvl-pathway inhibitor 3289-8625, or BIBF1120 blocking FGFR-signaling. Besides confirming gonad localization and receptor functions, inhibitor-induced phenotypes were observed *in vitro* such as decreased egg production as well as drastic effects on gonad differentiation, morphology, embryogenesis, and survival of adult worms. In summary, these results emphasise the usefulness of tissue-specific qRT-PCRs for selection of candidate genes with important roles in reproduction, allowing subsequent studies to determine their suitability as drug targets.

## Introduction

Besides their medical importance for humans and animals causing schistosomiasis (bilharzia), schistosome parasites exhibit a number of unusual features. Schistosomes possess a complex life-cycle, they belong to the trematodes but have evolved separate sexes, and their reproductive biology is governed by pairing-dependent processes (Popiel and Basch, 1984; Kunz, 2001; Chitsulo et al., 2004; King et al., 2005). In this context, a unique phenomenon of schistosome biology is that the female requires permanent pairing-contact with the male to become sexually mature. Without a pairing partner, the female remains sexually immature, containing stem cell-like precursor cells in the vitellarium and an ovary filled with immature oogonia. Following pairing, differentiation processes are induced leading to the complete differentiation of the vitellarium and the ovary, which contain mature S4-vitellocytes and primary oocytes, respectively. Both are needed for the formation of composite trematode eggs (Shaw and Erasmus, 1982; Popiel and Basch, 1984; Kunz, 2001). This is controlled by signal transduction processes and paralleled by a remarkable increase of the body size of the female (Knobloch et al., 2007; LoVerde et al., 2009; Beckmann et al., 2010). An improved understanding of the reproductive biology of this exceptional parasite is necessary, as the eggs are responsible for the pathology of schistosomiasis.

Since there is increasing fear of the development of resistance against the only commonly used drug applied to fight all schistosome species, Praziquantel (PZQ), and because there is no vaccine available yet, great efforts are being made to search for alternatives (Fenwick and Webster, 2006; Doenhoff et al., 2008; Melman et al., 2009). Among these are genomic, transcriptomic, and proteomic studies that have provided a huge amount of valuable information about the genetic repertoire of schistosomes (Verjovski-Almeida et al., 2003; Liu et al., 2006; Hokke et al., 2007; Berriman et al., 2009; Schistosoma japonicum Genome Sequencing and Functional Analysis Consortium, 2009; Protasio et al., 2012; Wilson, 2012; Young et al., 2012).

Now, in the dawn of the post-genomic era, methods are required to make use of the available genome data and to functionally characterize genes of interest. To this end we have established a protocol for the isolation of pure, intact testes and ovaries from adult schistosomes (Hahnel et al., 2013). Among other outcomes, it was demonstrated that gonad-specific RNA of high quality was obtained from each of these tissues allowing a detailed characterization of gene expression at the tissue level. In parallel, data on G protein-coupled receptors (GPCRs) were obtained (Zamanian et al., 2011; Hahnel et al., unpublished), and the influence of pairing on males was investigated by a combinatory transcriptomics approach using SuperSAGE and microarray hybridization (Leutner et al., 2013). These studies provided hints toward new genes that are influenced by pairing. Among these were gene homologs of the frizzled receptor SmFz1 (Smp_118970/173940), the membrane progestin receptor component 1 SmPMRC1 (Smp_093700), the RNA binding protein Musashi (Smp_157750), and the transmembrane receptor Notch (Smp_050520). Finally, a recent study identified neoblast-like stem cells embedded in somatic tissue in adult *S. mansoni*, and evidence was obtained for a role of a fibroblast growth factor receptor homolog, SmFGFR-A (Smp_175590), for the maintenance of these neoblast-like cells (Collins et al., 2013). Since the latter study provided additional evidence for SmFGFR-A transcripts in testes, this gene and a second member of this gene family in *S. mansoni*, SmFGFR-B (Smp_157300), were included in the following analysis.

To investigate the influence of pairing on the transcription of some of the above mentioned genes at the level of the gonads we performed comparative qRT-PCR analyses. Evidence was obtained that this approach allows the discovery of a pairing-influence on the transcription of some of these genes in the gonads, which is not detectable at the whole worm level. Furthermore, first functional characterization of SmFz1 and SmFGFR-A/B by molecular, biochemical, physiological, and morphological analyses confirmed their potential roles in gonad differentiation and reproduction. Based on the fatal phenotypes observed, our results using inhibitors against the Wnt/Fz-pathway (3289-8625) or against FGFR-signaling (BIBF1120) additionally identified both as interesting lead compounds. Here we show that the combination of organ isolation and detailed tissue-specific expression analyses offer new avenues for the identification and characterization of potential new drug targets, which are desperately needed in the fight against schistosomiasis.

## Materials and methods

### Ethics statement

All animal experiments were performed in accordance with the European Convention for the Protection of Vertebrate Animals used for experimental and other scientific purposes (ETS No 123; revised Appendix A) and have been approved by the Regional Council (Regierungspraesidium) Giessen (V54-19 c 20/15 c GI 18/10).

### Parasite maintenance

A Liberian strain of *Schistosoma mansoni* was maintained in *Biomphalaria glabrata* and in Syrian hamsters (*Mesocricetus auratus*) (Grevelding, 1995). Adult worms were obtained by hepatoportal perfusion of hamsters at day 42 post-infection. Unisexual worm populations were generated by monomiracidial intermediate-host infection as described elsewhere (Grevelding, 1995). Adult worms were transferred to Petri dishes of 60 mm diameter size containing 4 ml M199 medium (Sigma-Aldrich; supplemented with 10% Newborn Calf Serum (NCS), 1% HEPES [1 M] and 1% ABAM-solution [10,000 units penicillin, 10 mg streptomycin and 25 mg amphotericin B per ml]) in groups of either 20 couples, or 25 males, or 50 females per Petri dish and kept *in vitro* at 37°C and 5% CO_2_.

### Isolation of RNA and qRT-PCR analyses

Testes and ovaries of adult worms from bisexual and unisexual infections were isolated by a combined detergent/enzymatic-based approach as described in detail by Hahnel et al. (2013). Total RNA from adult schistosomes and gonad tissue was extracted using the PeqGOLD TriFast reagent (Peqlab) following the manufacturer's protocol. For this, 5–10 adult worms or 50–100 testes and ovaries, respectively, were incubated in 500 μl TriFast-solution. Pairs were separated by repeated pipetting immediately before processing. The adult worms were mechanically homogenized with a plastic piston whereas gonads were frozen in liquid nitrogen and thawed on ice three times to enhance tissue disintegration. Precipitation of total RNA in 2-propanol was aided by addition of 35 μg glycogen (RNase-free PeqGOLD glycogen, Peqlab). RNA quality and quantity were checked by electropherogram analysis employing the BioAnalyzer 2100 (Agilent Technologies). In brief, 1 μl of resuspended RNA was loaded on an Agilent RNA6000 Nano Chip according to the manufacturer's instructions and analyzed using the device setting “EukaryoteTotal RNA Nanoassay.”

Synthesis of cDNA was performed using the QuantiTect Reverse Transcription Kit (Qiagen) comprising a genomic DNA wipe out step and 500 ng of total RNA per reaction. The obtained cDNA was diluted 1:20 and used in subsequent qRT-PCR analyses. The detection of synthesized DNA double strands was based on the incorporation of SYBRGreen using PerfeCTa SYBR Green Super Mix (Quanta). To distinguish between specific amplification products and unspecific primer dimers following each qRT-PCR analysis, a melting point analysis was carried out. Primer 3 Plus software was used for primer design, and the amplification products had sizes between 140 and 160 bp. Primers were designed to have melting points at 60°C (Table S1) and were commercially synthesized (Biolegio, Netherlands). Amplification reactions were done in triplicate, and analyses were performed using a relative quantification against the reference gene actin (Smp_161930) with the Δ Δ Ct method (Livak and Schmittgen, 2001).

### Germinal vesicle breakdown (GVBD) assays in *Xenopus* oocytes

The tyrosine kinase domains of SmFGFR-A and SmFGFR-B were amplified by PCR using cDNA from adult worms as template. For the PCR reactions the following primers were used: 5′FGFR-B_TK-*Bam*HI: GGA TCC ATG AAA TGG TAT CTT CAG AGA GTC AAC AGC, 3′FGFR-B_TK-*Xba*I: TCT AGA TTC ACT AGT TTC AGT ACG ACC ATC and 5′FGFR-A_TK-*Bam*HI: GGA TCC GAA ATG GTT CAA CCA TCC AAA TAT TTT CCA CAG, 3′FGFR-A_TK-*Xho*I: CTC GAG TCC TTC AGG TCA CCA TAA CTG. All primers contained restriction sites at their 5′-end as indicated to allow an insertion of amplification products into the vector pcDNA3.1-B. The correct ORFs of the kinase constructs were confirmed by sequencing (LGC Genomics, Berlin). Plasmids were linearized by the restriction enzyme *Pme*I. Capped messengerRNA (cRNA) encoding the different TK domains were synthesized *in vitro* using the T7 mMessage mMachine kit (Ambion) and analyzed as described previously (Long et al., 2010). cRNA preparations were microinjected into *Xenopus laevis* stage VI oocytes according to a standard protocol (Vicogne et al., 2004). Each oocyte was injected with 60 ng of cRNA in the equatorial region and incubated at 19°C in ND96 medium. After 18 h, GVBD was detected by the appearance of a white spot at the center of the animal pole. For kinase inhibitor studies sets of 10 oocytes were freshly injected with SmFGFR-A or SmFGFR-B kinase domain constructs and placed in ND96 containing different concentrations of BIBF1120 (Vargatef, CAS-No. 656247-17-5; SelleckChem, dissolved in DMSO). GVBD was observed after 18 h. Non-injected oocytes served as negative controls. For positive controls, the natural hormonal stimulus progesterone was used (Sadler and Maller, 1983).

Dead kinase variants of SmFGFR-A and SmFGFR-B kinase domains (SmFGFR-A_TK-ko and SmFGFR-B_TK-ko) were generated by site-directed mutagenesis. Using the primer combination 5′FGFR-A_TK-ko: GGA TTT GTT GCA AAA TTA TGC GAT AAC GCT TAT GCA TGT ACC CAA GAG G/3′FGFR-A_K-ko: CCT CTT GGG TAC ATG CAT AAG CGT TAT CGC ATA ATT TTG CAA CAA ATC C and 5′FGFR-B_TK-ko: GGT AAA CAC TAT AAA TTA AAA ATT GCT GAT AAT GCA CTT ACA AGA TTT GCT GAA/3′FGFR-B_TK-ko: CCT CTT GGG TAC ATG CAT AAG CGT TAT CGC ATA ATT TTG CAA CAA ATC C, the Mg^2+^-binding motif DFG of the kinase domains was changed into a DNA motif, as described previously (Vicogne et al., 2004). In addition a constitutively active mutant of the FGFR-B_TK domain was produced by exchanging the lysine within the amino acid motif YYRK^519^ to a negatively charge glutamate (YYRE^519^). This mutation is consistent with the YYKE^650^ variation of the human FGF Receptor 3 (hFGFR3), which led to its ligand-independent activation (Neilson and Friesel, 1996; Webster et al., 1996). For the synthesis of FGFR-B_TK-active the following primer combination was used, 5′FGFR-B_TK-active: AGA TTT GCT GAA AAT TAT TAT CGT GAA ATG AAA AAT GGT CGT GTT CCG/3′FGFR-B_TK-active: CGG AAC ACG ACC ATT TTT CAT TTC ACG ATA ATA ATT TTC AGC AAA TCT.

### *In situ*-hybridization experiments

*In situ*-hybridizations were performed as described elsewhere (Quack et al., 2009; Buro et al., 2013). In short, adult worm pairs were fixed in Bouin's solution (picric acid/acetic acid/formaldehyde; 15:1:5, Sigma Aldrich) before embedding in paraplast (Histowax, Reichert-Jung). Sections of 5 μm were generated and incubated in xylol. Following rehydration, proteins were removed by proteinase K treatment (final concentration 1 μg/ml), and the sections were dehydrated. For hybridization, gene-specific transcripts were synthesized *in vitro* using T7 promoter-containing PCR products, and labeled with digoxigenin following the manufacturer's instructions (Roche Applied Science). For this approach the following primer pairs were used: SmFz1 (5′SmFz1_C-Term: GTG GTA AAA CGC TTG TAT CAT GG/ 3′SmFz1_C-Term: GTA AGC CTA GAC CAG AAT TAG C), SmFGFR-A (5′SmFGFR-A_insitu: GAT GAT GCA ATT AGA CAA CAA AGA G/ 3′FGFR-A_insitu: CGA TTA TCG GGA TCT TGT GAC), SmFGFR-B (5′SmFGFR-B_insitu: GTA TCT TCA GAG AGT CAA CAG C/ 3′SmFGFR-B_insitu: CGA TGA CGA CGC AGA TAC TC). To allow the synthesis of antisense or sense transcripts one primer of each reaction was tagged with the T7-sequence at its 5′-end. Labeled sense and antisense transcripts of SmFz1 (459 nt), SmFGFR-A (470 nt), and SmFGFR-B (437 nt) were size-controlled by gel electrophoresis. To prove their quality, transcript blots were made to confirm digoxigenin incorporation by alkaline phosphatase-conjugated anti-digoxigenin antibodies, naphthol-AS-phosphate, and Fast Red TR (Sigma Aldrich). All *in situ*-hybridizations were performed for 16 h at 42°C. Sections were stringently washed up to 0.5 × SSC, and detection was achieved as described for transcript blots.

### Inhibitor treatment of adult schistosomes

For *in vitro* culture experiments with inhibitors schistosome couples were transferred into supplemented M199 medium 24 h after perfusion. The inhibitors 3289-8625 (Dvl-PDZ Domain Inhibitor II, CAS-No. 294891-81-9; Merck Millipore) and BIBF1120 (Vargatef, CAS-No. 656247-17-5; SelleckChem) were dissolved in dimethyl sulfoxide (DMSO) and added in various concentrations to the culture medium. Control groups were cultured in medium containing DMSO only. Couples were kept *in vitro* at 37°C and 5% CO_2_, and medium and additives were refreshed daily.

### Morphological analyses

Egg development and worm morphology were monitored by bright-field microscopy (CX21, Olympus; Labovert FS, Leitz), and images were acquired by a digital camera (SC30, Olympus) with CellSens Dimension software (Olympus).

For further morphological analyses, adult worms were fixed for at least 24 h in AFA (ethanol 95%, formaldehyde 3%, and glacial acetic acid 2%), stained for 30 min with 2.5% hydrochloric carmine (CertistainH, Merck), and destained in acidic 70% ethanol. After dehydration in 70, 90, and 100% ethanol for 5 min each, worms were preserved as whole-mounts in Canada balsam (Merck) on glass slides (Neves et al., 2005). CLSM images were made on a Leica TSC SP2 microscope using a 488 nm He/Ne laser and a 470 nm long-pass filter in reflection mode as described before (Beckmann et al., 2010).

### EdU-incorporation assay

*S. mansoni* couples were treated with 5 μM of BIBF1120 or DMSO only for at least 48 h *in vitro*. After the first 24 h the medium was additionally supplied with 10 μM of thymidine analog 5-ethynyl-2′-deoxyuridine (EdU). EdU incorporation into adult schistosomes was detected essentially according to the manufacturer's instructions (Click-iT EdU Imaging Kit; Molecular Probes, Darmstadt, Germany), and as described by Collins et al. (2013). Briefly, treated schistosome couples were separated and subsequently fixed for 6 h in 4% paraformaldehyde in PBSTx (PBS with 0.3% Triton X-100). The worms were then rinsed once in PBSTx, before dehydration in 50% MeOH in PBSTx for 10 min at room temperature (RT) with shaking, followed by a 10 min in 100% MeOH. Parasites were stored in fresh MeOH at −20°C.

The worms were rehydrated through one 10 min wash each in 50% MeOH in PBSTx then PBSTx, after which they were treated with 6 μg/ml proteinase K for 25 mins at RT. The samples were post-fixed in 4% formaldehyde in PBSTx for 10 min at RT. After two washes in 3% BSA in PBS, the worms were incubated in Alexafluor 647 Click-iT reagent for 30 min with shaking. From this point on the samples were protected from light. Following the Click-iT reaction, the worms were washed twice in 3% BSA in PBS. Parasites were counterstained overnight at 4°C with 8 μM Hoechst 33342 in PBSTx. They were washed twice in PBSTx for 15 min at RT before being mounted on slides with Rotimount Fluorcare (Carl Roth, Karlsruhe, Germany). The specimens were viewed on a Leica TCS SP2 confocal microscope. Hoechst was excited with a 405 nm laser, and Alexafluor 647 was excited at 633 nm.

### *In silico* analyses

The following public domain tools were used: Gene DB (http://www.genedb.org/Homepage), Welcome Trust Sanger Institute S. mansoni OmniBlast (http://www.sanger.ac.uk/cgi-bin/blast/submitblast/s_mansoni/omni), NCBI BLAST (http://blast.ncbi.nlm.nih.gov/), SMART (http://smart.embl-heidelberg.de/). Primer 3 (http://www.bioinformatics.nl/cgi-bin/primer3plus/primer3plus.cgi).

## Results

### Influence of pairing contact on gene transcription in the gonads

In order to address the question whether the influence of pairing on transcription cannot only be seen at the whole worm level, but also in isolated gonads, we performed qRT-PCR analyses. To this end we compared relative transcript levels of selected genes between pairing-unexperienced (um) and pairing-experienced (em) *S. mansoni* males and females (uf/ef) as well as isolated testes and ovaries from such worms obtained using the recently established organ isolation protocol (Hahnel et al., 2013).

**Figure 1:**
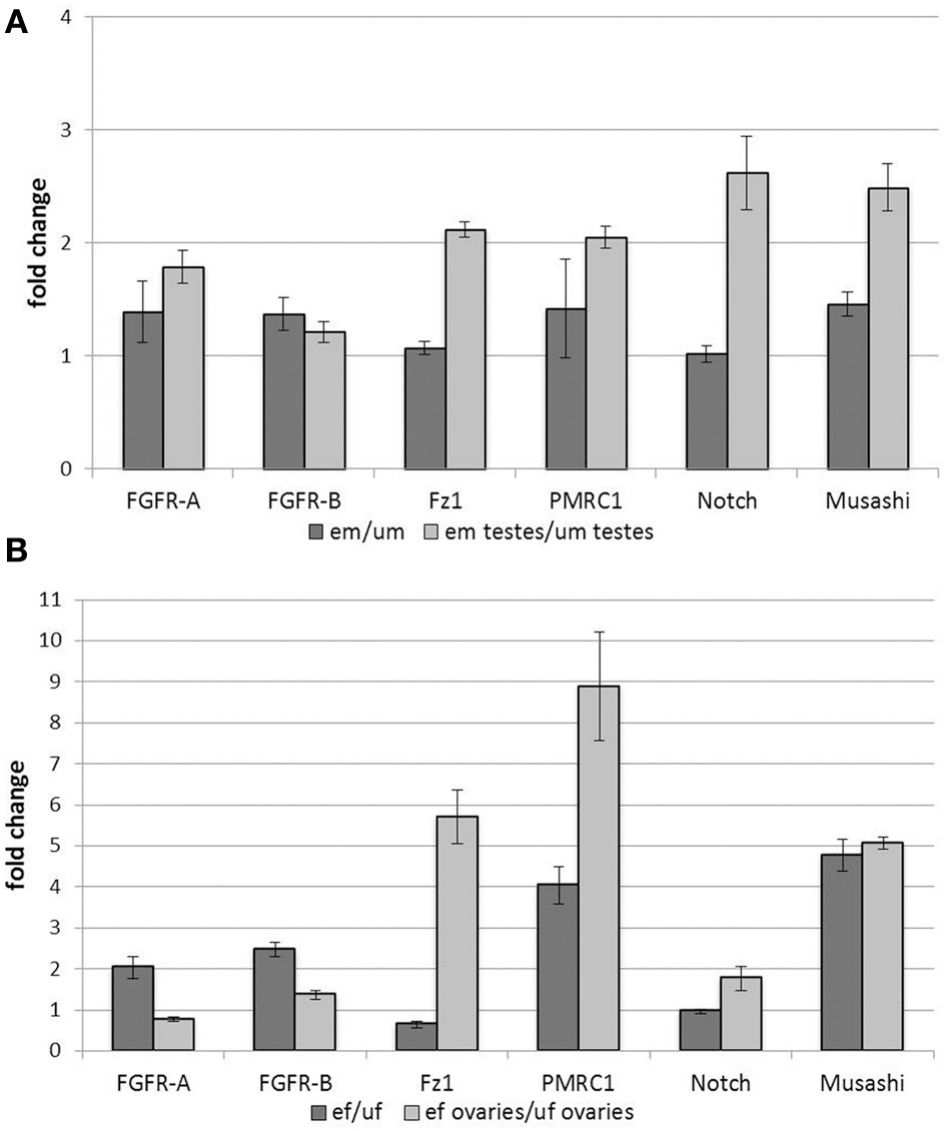
**Influence of pairing on gene transcription in adult worms and gonads**. Analyses of the influence of pairing on the transcription rates of candidate genes in whole worms and gonads. Transcription of SmFGFR-A, SmFGFR-B, SmPMRC1, and the musashi homolog were up-regulated in em compared to um, whereas SmFz1 and Notch were not differentially transcribed at the whole worm level. In contrast, focusing on the testes, all analyzed genes were transcribed more abundantly in the testes of em compared to those of um **(A)**. Comparably, in female worms, most of the candidate genes were up-regulated following pairing with the exception of Notch and SmFz1, which appeared not differentially and less transcribed in ef compared to uf, respectively. Focusing on ovary-specific gene expression, transcription of SmPMRC1, Musashi as well as SmFz1 were strongly up-regulated by pairing. Compared to this, pairing led to a less remarkable effect on the expression the two FGFR-homologs and Notch in the female gonad **(B)**. In both diagrams the statistical evaluation of three technical replicates is shown (error bars indicated).

Most of the analyzed genes showed an up-regulation of transcription upon pairing at the levels of whole male worms (Figure 1A). This included genes encoding the two FGFR homologs SmFGFR-A and SmFGFR-B as well as a homolog of the progestin membrane receptor component 1 SmPMRC1 (Smp_093700), and a homolog of the RNA-binding protein Musashi (Smp_157750). In contrast, transcription rates of a Notch receptor homolog (Smp_050520) and the frizzled receptor SmFz1 seemed not to be affected by pairing. Focusing on the male gonads, however, all analyzed genes were transcribed more abundantly in the testes of em compared to those of um. With respect to the testes, especially SmFz1, the Notch homolog, and Musashi showed the highest up-regulation in their transcript levels upon pairing contact. These findings revealed pairing-dependent differences in transcriptional activity in the gonads which were not apparent at the whole-worm level.

In female worms, most of the genes investigated were up-regulated by pairing with the exception of SmFz1 and Notch, which seemed to be slightly down-regulated or non-regulated, respectively (Figure 1B). Again, tissue-specific analyses revealed paring-dependent transcription regulation in the ovary, which was not always apparent at the whole worm level. Besides SmPMRC1 and musashi, which were both also strongly influenced by pairing in the ovary, SmFz1 was found to be strongly up-regulated in this organ. Compared to this, pairing led to a less remarkable effect on the expression of the two FGFR-homologs and Notch in the female gonad.

### Sequence analyses of SmFz1, SmFGFR-A and SmFGFR-B

Searching the genome of *S. mansoni* for frizzled genes using the database GeneDB (Protasio et al., 2012) an annotated entry (Smp_118970) was found coding for the C-terminal fragment of a frizzled homolog named SmFz1. Full-length cloning of SmFz1 revealed that the missing N-terminal part was coded by another sequence annotated as Smp_173940. The whole open reading frame (ORF) of the frizzled gene consists of 2091 bp and an alignment with the GeneDB entries Smp_119870/Smp_173940 revealed a sequence identity of 99% (accession number: KJ820759).

**Figure 2:**
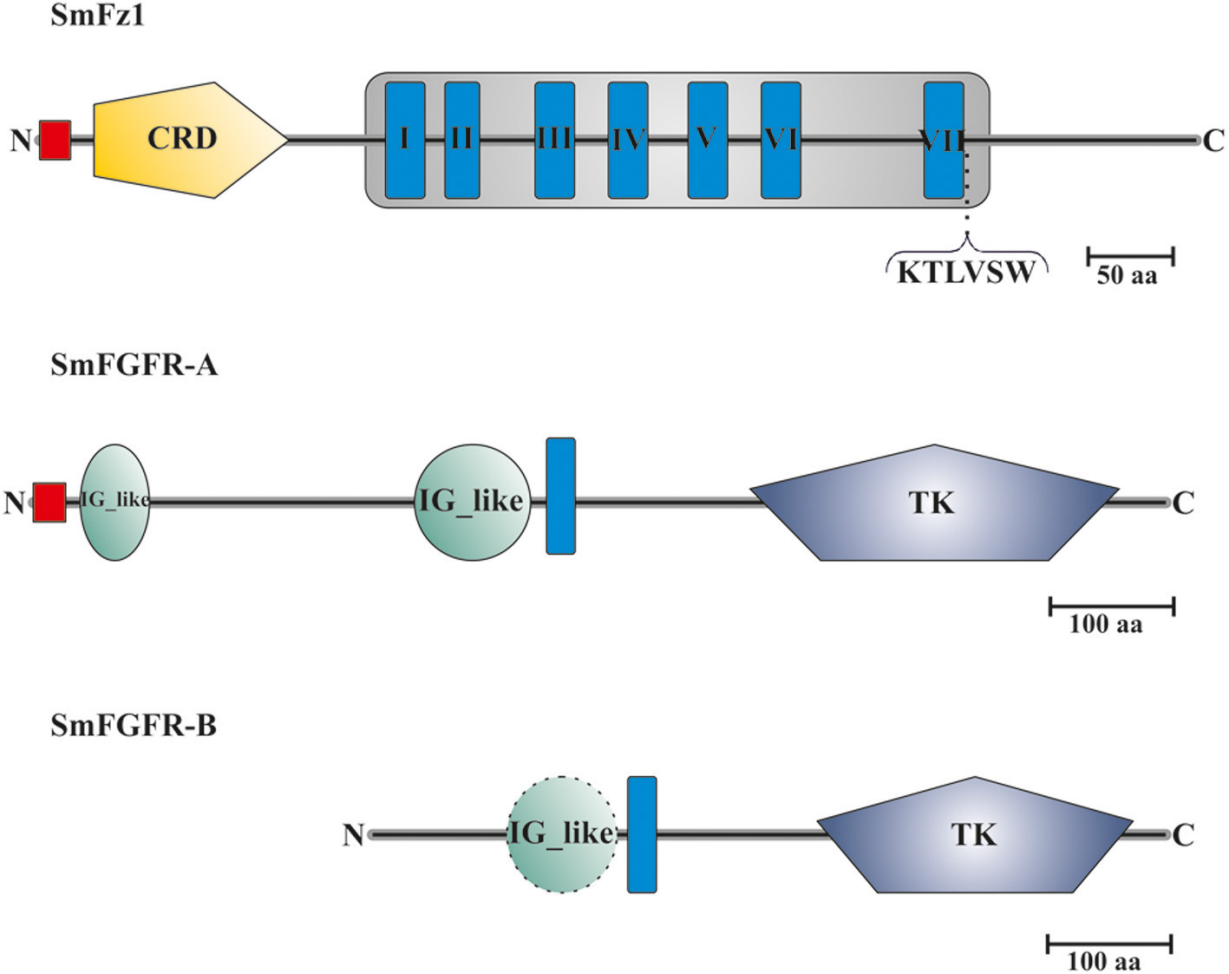
**Domain structure analyses of SmFz1, SmFGFR-A, and SmFGFR-B**. Schematic structures of the frizzled homolog SmFz1, and the two FGFR homologs SmFGFR-A and SmFGFR-B from *S. mansoni*. SmFz1 comprises an N-terminal signal peptide sequence (red) and an extracellular CRD domain (yellow) sufficient for Wnt ligand binding. The frizzled transmembrane domain (gray box) contains seven TMHs (I–VII). Following TMH VII, a Dvl-binding motif (KTLVSW) is located at the beginning of the intracellular C-terminus. Both FGFRs possess TK domains within their intracellular C-termini but differ in the structures of their extracellular parts (TMHs in blue). SmFGFR-A consists of an N-terminal signal peptide sequence (red) followed by two IG-like domains (green) sufficient for ligand binding **(A)**. In contrast the N-terminus of SmFGFR-B is smaller in size and contains only one putative IG-like domain (dashed line), which was not rated as significant **(B)**.

Sequence analyses showed that the predicted protein sequence of SmFz1 contained typical features of frizzled receptors, which belong to the superfamily of seven transmembrane receptors (Schiöth and Fredriksson, 2005) (Figure 2). A signal peptide of 18 amino acids length is located at the N-terminus (M^1^ to C^18^), which is probably involved in the localization of the receptor to the plasma membrane. This motif is followed by an extracellular cysteine-rich domain (CRD) (K^33^ to K^147^) sufficient for the binding of Wnt ligands (Schulte, 2010). The frizzled transmembrane domain extends from L^193^ to R^560^ and contains seven transmembrane helices (THMs). The seventh TMH is followed by the amino acid motif K^545^TLVSW. This corresponds to the conserved Disheveled (Dvl) binding motif consensus sequence KTXXXW located at the beginning of the intracellular C-terminus of frizzled receptors (Wong et al., 2003).

The genome of *S. mansoni* encodes two FGFR homologs named SmFGFR-A (Smp_175590) and SmFGFR-B (Smp_157300). A detailed *in silico* analysis showed that SmFGFR-A consists of 918 amino acids (Figure 2). The extracellular N-terminus starts with a signal peptide sequence (M^1^–G^26^) followed by two extracellular immunoglobulin(IG)-like domains located at the positions T^39^ to S^94^ and S^310^ to M^403^. IG-like domains are common features of FGFRs and are involved in ligand binding and auto-regulation processes (Eswarakumar et al., 2005). The single TMH is located from position P^417^ to I^439^. The intracellular C-terminus contains a catalytic tyrosine kinase (TK) domain (F^582^ to I^880^), which is involved in downstream signaling events (Eswarakumar et al., 2005).

In contrast to SmFGFR-A, the N-terminal signal peptide as well as characteristic IG-like domains are missing within the SmFGFR-B protein sequence (643 aa) (Figure 2). At position P^101^ to N^189^ a putative IG-like domain was identified, but the degree of homology was not significant according to SMART. The single TMH is located at position W^199^ to W^221^ followed by the catalytic tyrosine kinase domain (L^353^ to L^617^).

### Localization of SmFz1, SmFGFR-A and SmFGFR-B transcripts by *in situ*-hybridization

**Figure 3:**
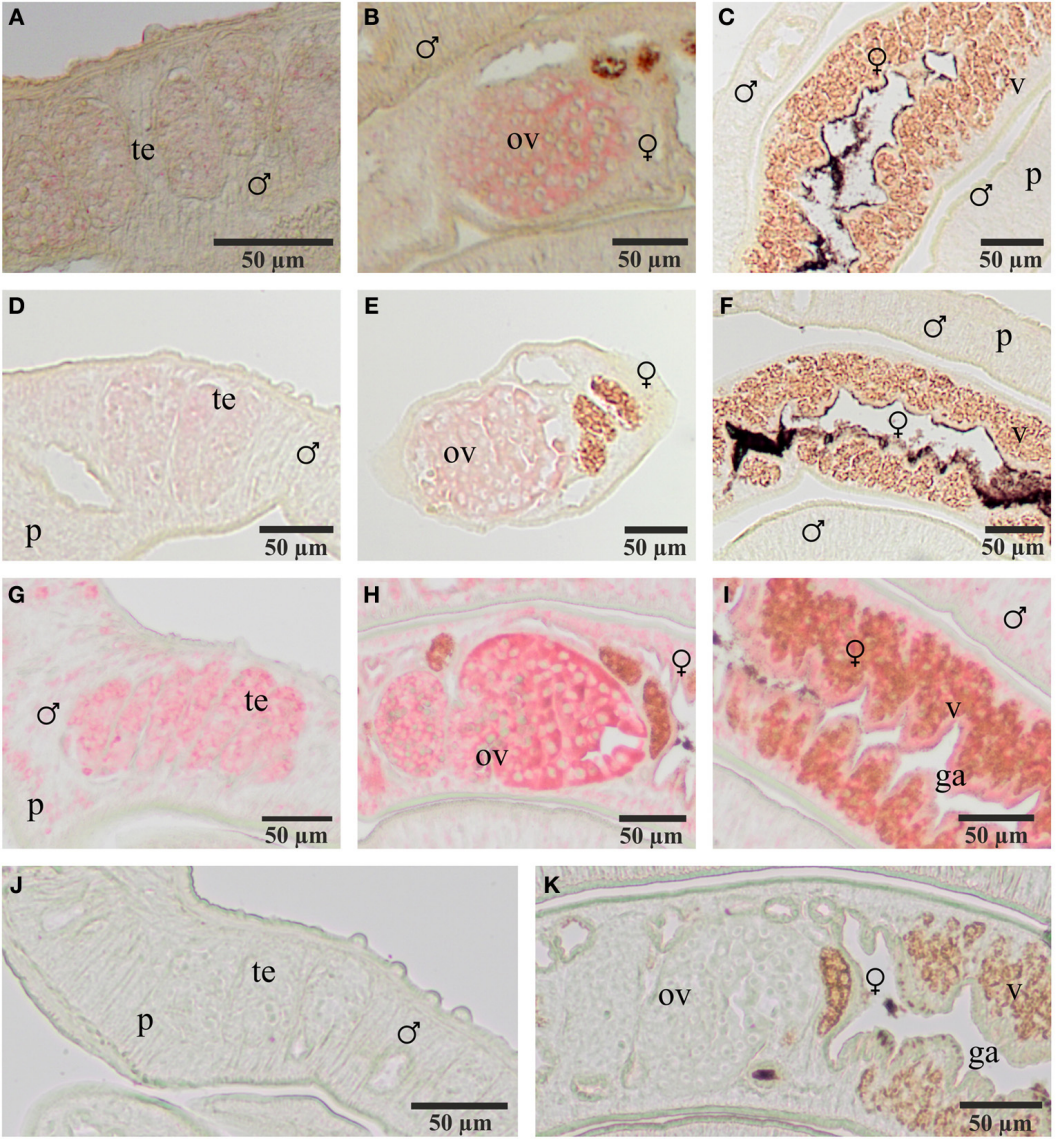
**Localization of transcripts of SmFz1, SmFGFR-A, and SmFGFR-B in adult *S. mansoni***. Results of *in situ*-hybridization experiments to localize transcripts of SmFz1 **(A–C)**, SmFGFR-A **(D–F)**, and SmFGFR-B **(G–I)** using DIG-labeled antisense-RNA probes on 5 μm sections of *S. mansoni* couples. Transcripts of all genes were detected in the testes of the male and the ovary of the female. SmFGFR-B transcripts were also observed in the vitellarium of the female and the parenchyma as well as the gastrodermis of both genders. Negative controls using sense-RNA probes showed no color reaction **(J,K)**. [te, testes; ov, ovary; v, vitellarium; p, parenchyma, g, gastrodermis; ♂, male; ♀, female].

To localize transcripts of SmFz1 and the FGFR homologs SmFGFR-A and SmFGFR-B in schistosomes *in situ*-hybridization experiments were performed on sections of *S. mansoni* couples (Figure 3). As predicted by the gonad-specific qRT-PCR experiments, the occurrence of transcripts of all three transmembrane receptors was confirmed in the testes of males and in the ovaries of females. In addition, SmFGFR-B transcripts were detected in the vitellarium of the female and in the gastrodermis as well as the parenchyma of both genders. For SmFGFR-A weak signals also occurred in the parenchyma (data not shown), which is in accordance with former whole mount *in situ* hybridization studies (Collins et al., 2013).

### Blocking of Frizzled-Dvl signaling led to dramatic phenotypes in the gonads and inhibited egg embryogenesis

To investigate the role of Frizzled-signaling in reproduction and developmental processes of schistosomes, *S. mansoni* couples were treated *in vitro* with different concentrations of the commercially available compound 3289-8625 (also Dvl-PDZ domain inhibitor II, Merck Millipore). This inhibitor blocks canonical Frizzled-signaling by binding to the PDZ (post-synaptic density-95/discs large/zonula occludens-1) domain of the Frizzled downstream interaction partner Disheveled (Dvl) with an IC_50_ of 12.5 μM in cell culture experiments (Grandy et al., 2009; Voronkov and Krauss, 2013). Because of the drug's specificity targeting the Frizzled-Dvl interaction directly we performed database analyzes to identify Dvl homologs in the genome of *S. mansoni*. As results two Dvl genes SmDvl1 (Smp_162410) and SmDvl2 (Smp_020300) were found and their transcription in testes and ovaries was confirmed by gonad-specific RT-PCRs (data not shown).

**Figure 4:**
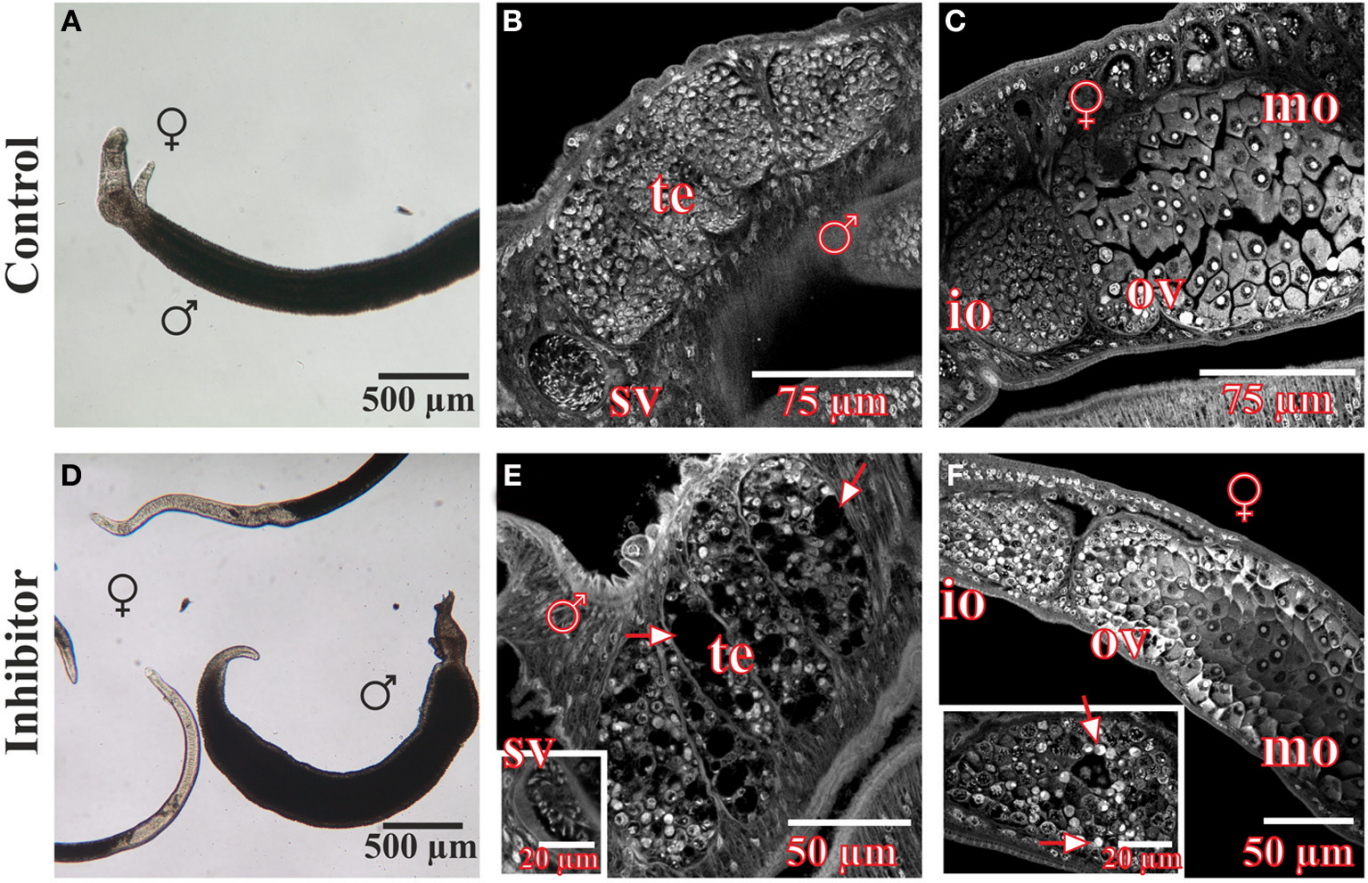
**Morphology of *S. mansoni* couples after treatment with the Frizzled-Dvl inhibitor 3289-8625 *in vitro.*** *S. mansoni* couples were cultured *in vitro* for 96 h with different concentrations of the Frizzled-Dvl inhibitor 3289-8625 and subsequently studied by bright field microscopy and CLSM for morphological changes. Whereas no morphological alterations were seen in control worms **(A–C)**, inhibitor treatment (500 μM) led to a reduced vitality accompanied by a separation of couples **(D)**. Furthermore, CLSM-analyses revealed severe effects of the inhibitor on the morphology of the gonads. The testes of treated males contained large pore-like structures (**E**, arrows) whereas ovaries of females were mostly affected in the anterior part containing several damaged cells and cell fragments (**F**, arrows). [te, testes; sv, seminal vesicle; ov, ovary; io, immature oocytes; mo, mature oocytes; ♂, male; ♀, female].

*In vitro*-culture experiments with inhibitor concentrations up to 400 μM for at least 96 h had no significant effect on the vitality, morphology, pairing stability or egg production of treated worms (data not shown). Only 500 μM or higher concentrations of 3289-8625 led to obvious alterations of worm morphology and survival within 24 h (Figure 4). Compared to control couples treated worms separated during this period, and male worms were no longer attached to the Petri dish. Using confocal laser scanning microscopy (CLSM) more detailed analyses revealed drastic influences of the drug on the morphology of the reproductive organs of both genders. The testes of treated males contained large, pore-like structures whereas in the ovaries of females the greatest effects were observed in the anterior part which contained numerous damaged cells and cell fragments (Figure 4).

**Figure 5:**
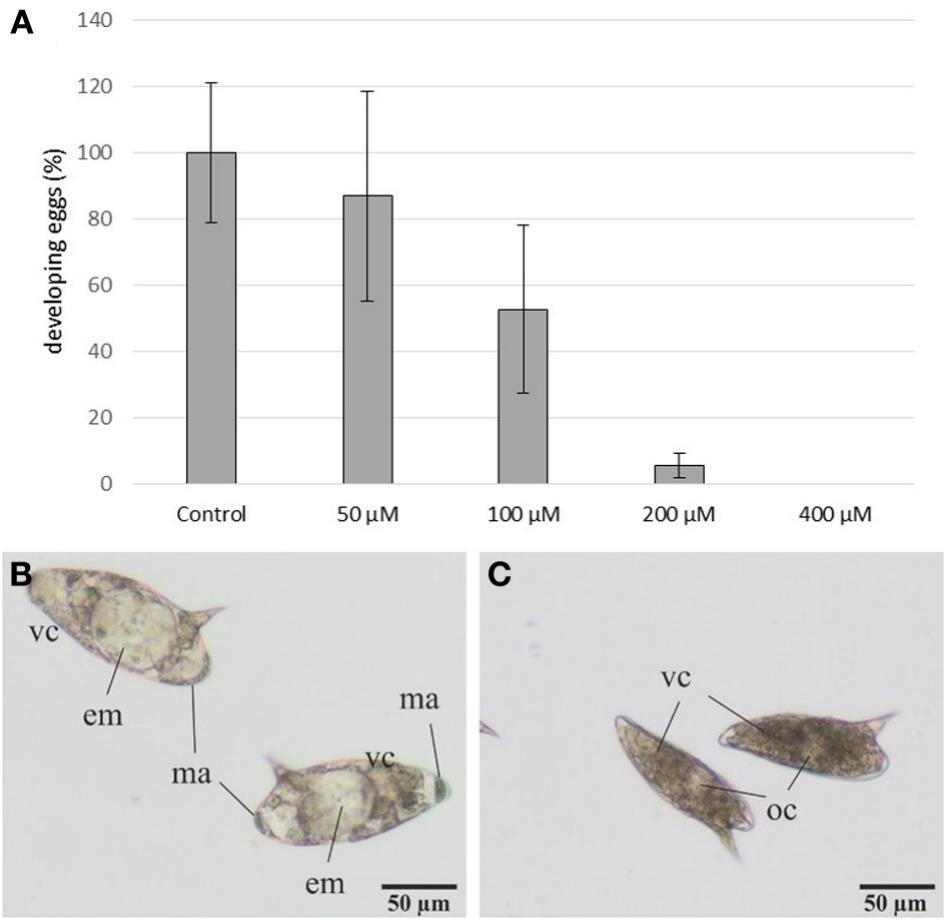
**Influence of the inhibitor 3289-8625 on the development of *S. mansoni* eggs**. *S. mansoni* couples were treated with different inhibitor concentrations *in vitro*. After 24 h worms were removed from the dishes, and the deposited eggs were cultured for an additional 3-4 day period. Subsequent analyses revealed a concentration-dependent effect of 3289-8625 on egg development compared to the DMSO control **(A)**. The statistical evaluation of three independent experiments is shown (error bars indicated). Untreated eggs showed continued development and were categorized into the stages II and III of embryogenesis as defined by Jurberg et al. (2009) **(B)**. Upon increasing inhibitor concentrations embryogenesis was remarkably affected resulting in a larger proportion of undeveloped eggs **(C)**. [em: embryo; ma: macromeres; oc: oocyte; vc: vitelline cells].

Eggs were more susceptible to 3289-8625 treatment. Eggs laid by treated worms were cultured for 3–4 days (at 37°C and 5% CO_2_) and observed by bright-field microscopy (Figure 5). At this time point, control eggs had reached stages II and III according to the definition provided by Jurberg et al. (2009). This is evidenced by the increased size of both the egg and the embryo, which fills 50–70% of the egg and is transparent in appearance. Macromeres are present at the poles; these will become the outer envelope of the miracidium (Jurberg et al., 2009). The number of eggs reaching this developmental stage decreased with increasing inhibitor concentration (Figure 5). At 100 μM only 50% of the eggs developed, whilst at concentrations of 200 μM or higher, less than 10% reached stage II. The immature eggs were smaller and appeared brownish in color, probably due to an interrupted degradation of vitelline cells.

### GVBD assays confirmed the kinase activities of SmFGFR-A and SmFGFR-B and their inhibition by BIBF1120

To get first hints for the functions of FGFR-signaling in the reproduction biology of adult schistosomes, *S. mansoni* couples were treated *in vitro* with the commercially available angiokinase inhibitor BIBF1120 (Hilberg et al., 2008; Roth et al., 2009) for at least 4 days. This compound specifically blocks the enzymatic activity of human vascular endothelial growth factor (VEGF)-, platelet derived growth factor (PDGF)- and FGF-receptors with IC_50_ values between 20 and 100 nM in enzymatic assays by binding to the ATP-binding site of the kinase domains of RTKs in cell cultures (Hilberg et al., 2008; Roth et al., 2009). Using BLAST analyses no homologs of VEGFRs and PDGFRs were found in the genome of *S. mansoni.* Thus, SmFGFR-A and SmFGFR-B are likely to represent the major targets of this inhibitor in schistosomes.

To test the activity of BIBF1120, we expressed the TK domains of the two schistosome FGFR homologs in *Xenopus* oocytes and performed germinal vesicle breakdown (GVBD) assays. This system was successfully used to express schistosome TKs and to monitor their enzymatic activity under the influence of kinase inhibitors (Long et al., 2010; Beckmann et al., 2011; Vanderstraete et al., 2013). To induce GVBD in oocytes it is necessary to express an active form of the kinase. For this reason a constitutively active variant of the SmFGFR-B TK domain was generated by site-directed mutagenesis, in which the lysine of the sequence motif YYRK^519^ was changed to glutamate (YYRE^519^). This amino acid exchange corresponds to the YYKK^650^ to YYKE^650^ mutation in the activation loop of the human (h)FGFR3, which led to a ligand-independent receptor activation (Neilson and Friesel, 1996; Webster et al., 1996) (Figure S1). In (h)FGFR3 phosphorylation of both regulatory tyrosine residues N-terminally of K^650^ unblock the catalytic site of the enzyme. The K^650^E mutation mimics this phosphorylation by introducing a negative charge and induces conformational changes which allow access to the catalytic site. In the wild type form of the receptor these conformational changes occur upon ligand binding-induced receptor dimerization, followed by trans-phosphorylation of the aforementioned tyrosines. Comparable mutations have also led to constitutively active variants of other RTKs including the human insulin receptor (Hubbard et al., 1994) and the schistosome venus kinase receptors (VKRs) SmVKR1 and SmVKR2 (Ahier et al., 2009; Beckmann et al., 2011; Gouignard et al., 2012). As expected the expression of FGFR-B_TK-active but not that of FGFR-B_TK-wt was sufficient to induce GVBD in *Xenopus* oocytes (Table 1).

**Table 1:** **Influence of BIBF1120 on the capacity of the TK domains of FGFR-A and FGFR-B to induce GVBD in *Xenopus* oocytes**.

**BIBF1120**	**0μM**	**0.5μM**	**1μM**	**2μM**	**5μM**	**10μM**
FGFR-A_TK-wt	100	100	100	82.5	0	0
FGFR-B_TK-active	100	100	70	0	0	0

*FGFR-TK induced GVBD (numbers represent % GVBD; mean of two independent experiments) in Xenopus oocytes was completely blocked by BIBF1120 at concentrations of 2 and 5μM, respectively*.

Interestingly, alignments of several FGFR kinase domains revealed that SmFGFR-A differs from other receptors with respect to the conserved consensus sequence YY(K/R)K of the activation loop. In contrast, FGFR-A has the motif GYME^781^ and contains a negatively charged glutamate C-terminally of the presumptive regulatory tyrosine residue. This raised the question of how the enzymatic activity of the TK domain is regulated and suggested the possibility that the native receptor is *per se* constitutively active. To obtain evidence for this hypothesis, the wild type TK domain of SmFGFR-A was expressed in the *Xenopus* system and induced GVBD in oocytes to nearly 100%. The GVBD-inducing capacity of the TK domains of SmFGFR-B_TK-active and SmFGFR-A_TK-wt was completely blocked at BIBF1120 concentrations of 2 μM and 5 μM, respectively (Table 1).

### BIBF1120 had significant effects on adult worms *in vitro*

**Figure 6:**
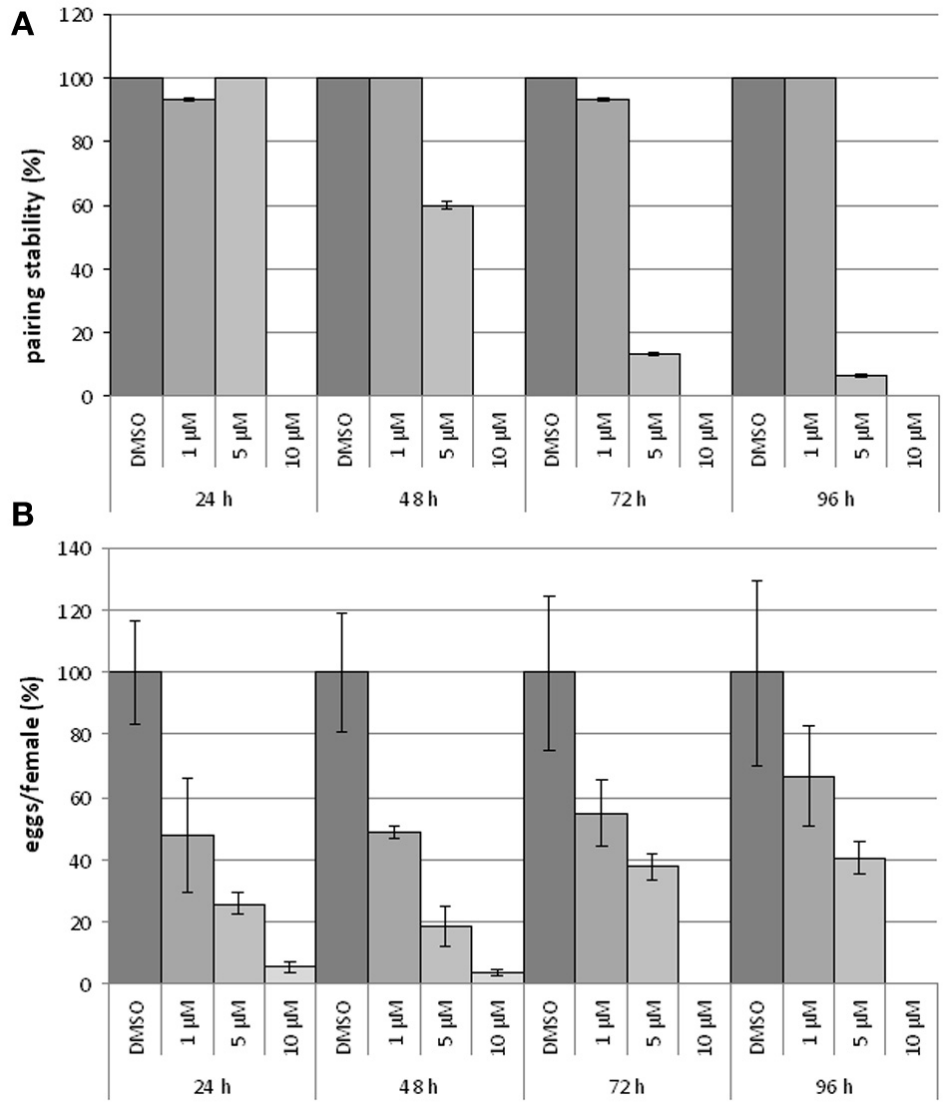
**Influence of BIBF1120 on pairing stability and egg production on *S. mansoni* couples *in vitro***. Treatment of *S. mansoni* couples with BIBF1120 for 96 h *in vitro* revealed a concentration-dependent effect of the FGFR inhibitor on pairing stability **(A)** and egg production **(B)** compared to the control (DMSO). Whereas 1 μM BIBF1120 had no influence on pairing, couples treated with 5 μM separated during the experiment period. The highest inhibitor concentration (10 μM) supplied, led to a separation of all couples within the first 24 h **(A)**. An obvious effect of BIBF1120 on egg production was also detected after 24 h. At this time point the inhibitor treatment led to a decrease of eggs down to 50% (1 μM) and to 20% (5 μM) compared to the control. At higher concentrations egg production was nearly completely disrupted **(B)**. In both diagrams the statistical evaluation of three independent experiments is shown (error bars indicated).

To study the influence of BIBF1120 on the reproduction and vitality of adult schistosomes, couples were treated with different inhibitor concentrations (1, 5, and 10 μM) *in vitro* for at least 96 h; egg production as well as pairing stability were determined daily (Figure 6). A drug concentration of 10 μM led to severe effects on worm morphology and survival. Within the first 24 h couples separated, and the worms were no longer attached to the bottom of the Petri dish. After 48 h treated worms showed extensive gut swelling and a curled body shape. At this time point other signs of viability such as gut peristalsis, muscular activity as well as egg production had almost completely stopped. The use of 5 μM BIBF1120 led to similar phenotypes, however, a time delay in the inhibitor effect was observed. Half of the treated couples started to separate within 48 h and 1 day later only 10% of the couples remained paired. An even more dramatic effect was observed on the reproductive capacity as egg production declined to about 20% compared to the control in the first 24 h. Alterations of the gut as well as general restriction of the viability were observed between 48 h and 72 h following treatment.

With respect to the influence of BIBF1120 on schistosome reproduction, the most interesting results were those obtained by supplying 1 μM of the inhibitor to *S. mansoni* couples. Treated couples stayed stable during the whole experiment, and worms showed no morphological alterations. Nevertheless, compared to the controls egg production was already reduced to less than 50% after 24 h treatment.

### CLSM analyses exhibited severe morphological effects in BIBF1120-treated *S. mansoni* couples

**Figure 7:**
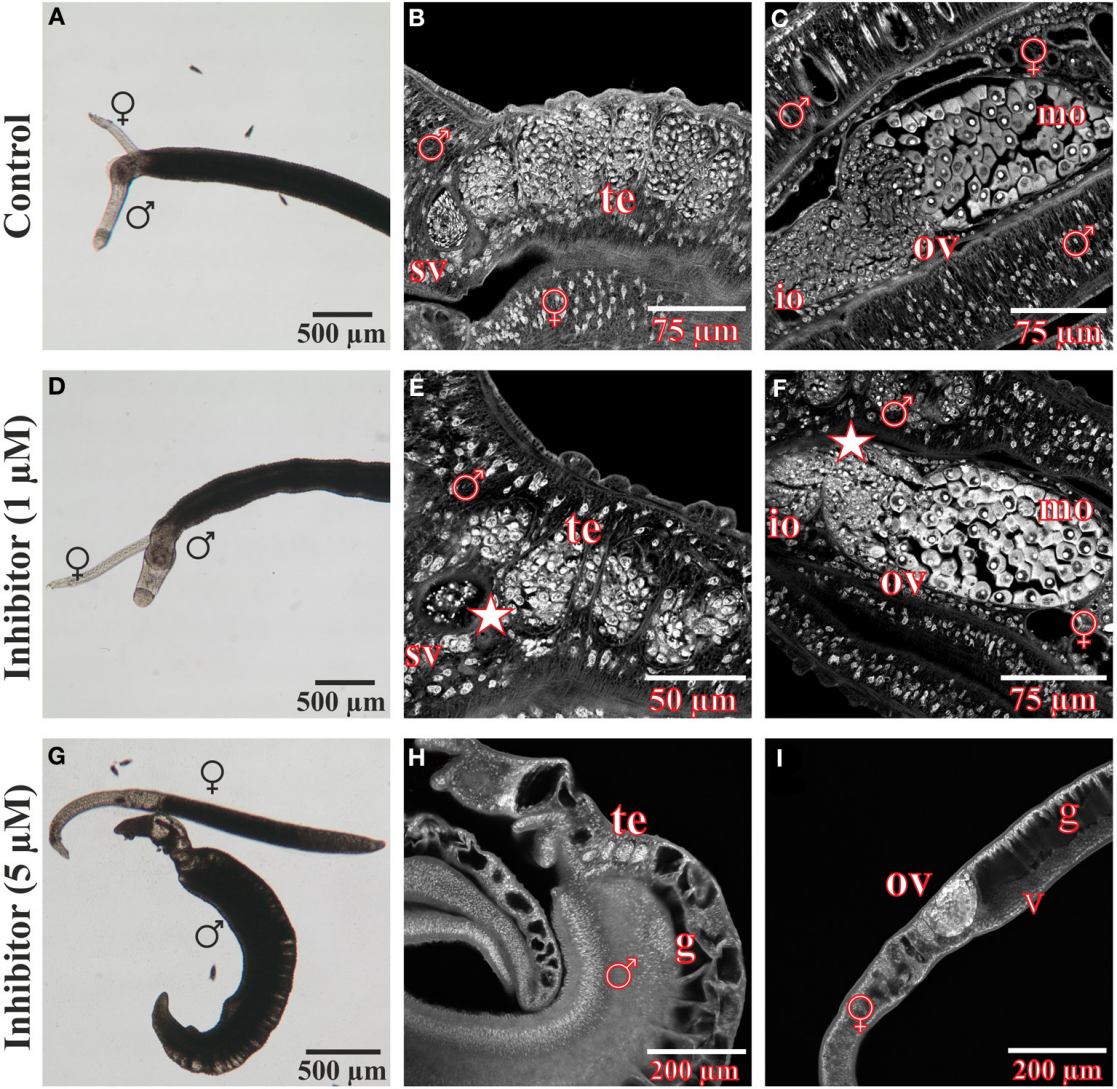
**Morphological analyses of *S. mansoni* couples after *in vitro* treatment with the inhibitor BIBF1120**. Couples of *S. mansoni* were treated with different concentrations of the FGFR inhibitor BIBF1120 for 96 h *in vitro* **(D–I)** und subsequently analyzed for morphological alterations. Worms supplied with 1 μM inhibitor showed no obvious changes in vitality and morphology compared to those of the DMSO control using bright field microscopy **(A,D)**. A more detailed examination by CLSM revealed an effect of BIBF1120 treatment on the gonads of both genders. Testicular lobes of treated males were reduced in their diameter and the seminal vesicle contained fewer mature sperms (**E**, asterix). In females 1 μM of inhibitor led to a partial degradation of immature oocytes in the anterior part of the ovary (**F**, asterix) (DMSO control: **B,C**). In contrast 5 μM BIBF11120 had severe effects on worm vitality and pairing stability as well as morphology in general **(G–I)**. Those worms showed a dramatically swollen intestinal tract and cells of the gonads were severely damaged. [te, testes; sv, seminal vesicle; ov, ovary; io, immature oocytes; mo, mature oocytes v, vitellarium; ga, gastrodermis; ♂, male; ♀, female].

After treatment for 96 h with the FGFR inhibitor BIBF1120, *S. mansoni* couples were analyzed in more detail using CLSM to investigate the influence of the compound on worm morphology (Figure 7). Compared to the DMSO control, gonad morphology was influenced following treatment with 1 μM BIBF1120, a concentration at which no inhibitor-phenotypes were observed by light microscopy. Testicular lobes of males were smaller in diameter, and seminal vesicles rarely contained mature sperms. The anterior part of ovaries in treated females contained a mass of degenerated oocytes which were not clearly distinguishable from each other.

As already observed by bright-field microscopy, an inhibitor concentration of 5 μM exerted severe effects on worm morphology and viability. Separated males and females had dramatically swollen guts. Cells of the gonads were also severely affected and appeared damaged.

### EdU-incorporation of BIBF1120-treated *S. mansoni* couples

**Figure 8:**
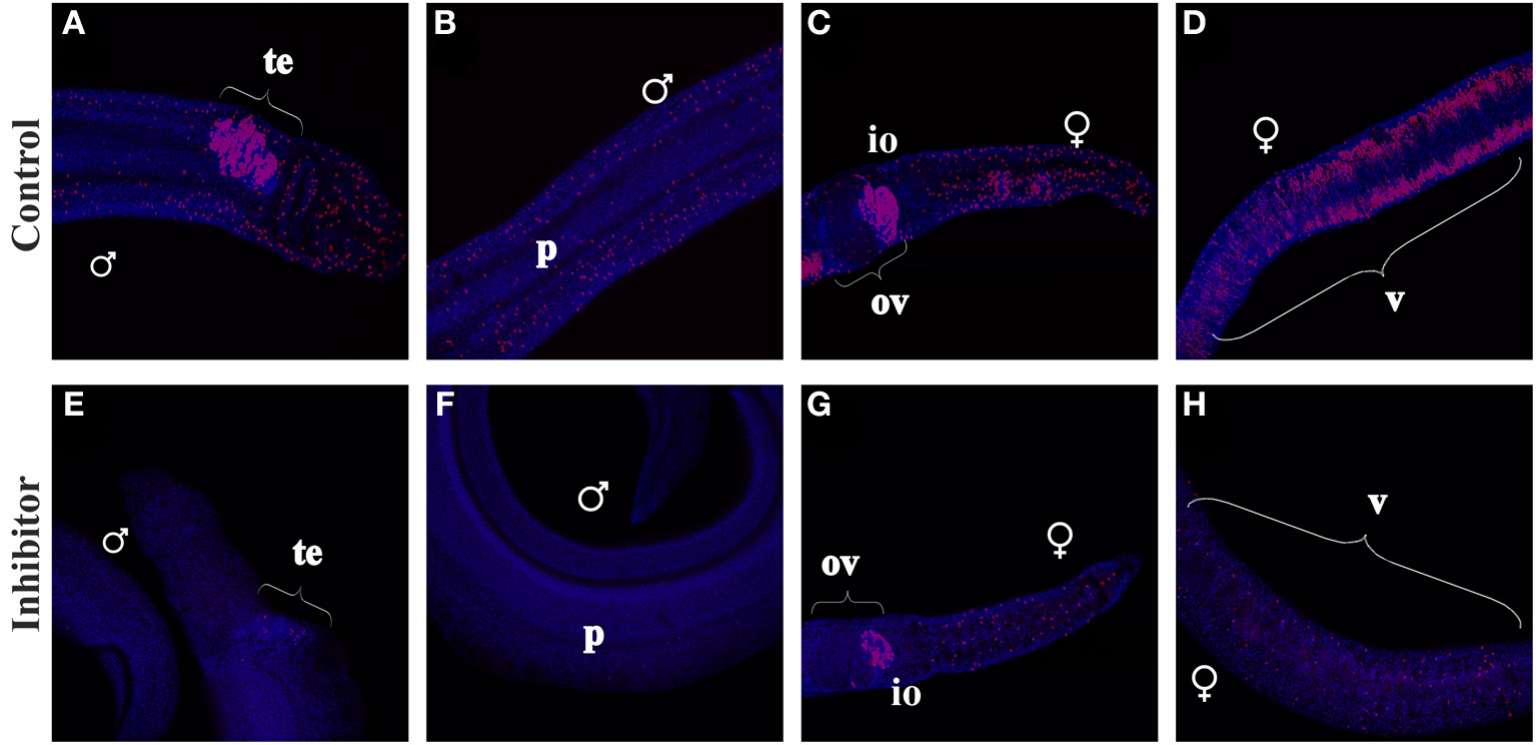
**EdU-incorporation of BIBF1120-treated *S. mansoni* couples**. *S. mansoni* couples were co-cultured *in vitro* for 48 h with BIBF1120, with the addition of EdU after 24 h, to investigate the influence of the inhibitor on mitotically active cells. In worms of the control group EdU^+^-cells were detected in the parenchyma and the gonads of both genders as well as in the vitellarium of the female **(A–D)**. Application of 5 μM BIBF1120 led to a drastic decline of EdU^+^-cells in all tissues, although the effect on the ovary was the weakest **(E–H)**. [te, testes; ov, ovary; io, immature oocytes; v, vitellarium; p, parenchyma; ♂, male; ♀, female].

In a previous study, FGFR-signaling has been linked to somatic stem cell control in adult schistosomes (Collins et al., 2013). To investigate whether FGFR-signaling also plays a role in reproductive organs, *S. mansoni* couples were cultured with EdU-containing medium in the absence or presence of BIBF1120 aiming to investigate its potential effect on mitotically active cells with stem-cell characteristics (Figure 8). A large number of EdU-labeled cells were detected in males and females of the control group. Signals were observed in the vitellarium and the anterior part of the ovary, which contain the stem cell-like S1-vitellocytes and the oogonia, respectively. This indicated high mitotic activity in these organs, which was expected since this is a prerequisite for paired, sexually mature females to produce high amounts of S4-vitellocytes and primary oocytes for egg production (Erasmus, 1973; Popiel and Basch, 1984; Kunz, 2001; Knobloch et al., 2002, 2007).

In males high amounts of mitotically active cells were identified in the testes (Figure 8), which certainly represent spermatogonia. Furthermore, EdU^+^-cells were detected within the parenchyma of both genders. In contrast inhibitor-treated worms showed a drastic decline of EdU^+^-cells in those tissues except of the ovary, which seemed to be less affected than other organs.

## Discussion

In the past, several studies have focused on the identification and characterization of biological processes involved in the male-induced maturation of the female, as this is a prerequisite for egg production. These efforts included global transcriptomic approaches providing broad insights into pairing-regulated gene expression in male and female *S. mansoni* (Fitzpatrick and Hoffmann, 2006; Waisberg et al., 2007; Williams et al., 2007; Leutner et al., 2013). Nevertheless, due to the restricted access to inner organs and structures, it has not been possible so far to study the influence of pairing on tissue-specific gene expression, especially in the reproductive organs. To overcome this restriction we recently established a protocol for the isolation of testes and ovaries from adult schistosomes (Hahnel et al., 2013).

Based on this approach, in the present study comparative qRT-PCR analyses were performed to investigate the influence of pairing on the gonad-specific expression of selected genes, which are presumably involved in developmental and differentiation processes. These included genes coding for different types of transmembrane proteins like a Notch receptor homolog, the frizzled receptor SmFz1, two FGFRs (SmFGFR-A and SmFGFR-B), as well as the membrane progestin receptor component SmPMRC1. Additionally, a Musashi homolog was included in the analyses. As a RNA-binding protein, in *Drosophila* Musashi controls among other functions, translational events in the germ line (Gunter and McLaughlin, 2011).

Determining the relative transcription levels of these genes in whole worms revealed their expression in adult *S. mansoni* of both genders independently of the pairing status. Additionally, SmFGFR-A, SmFGFR-B, SmPMRC1, and Musashi were up-regulated in males and females by pairing providing evidence for their roles in biological processes related to pairing and sexual maturation.

In addition, at the level of the gonads all investigated genes were found to be transcribed in testes and ovaries independently of the pairing status. This indicated their involvement in biological functions in these reproductive organs. Comparing transcript amounts in testes of um and em, we discovered that all genes analyzed were up-regulated upon pairing. For em this may be explained by an increased spermatogenesis. In a former morphological study it was described that testicular lobes of em contain more cells than those of um (Neves et al., 2005). In ef gonads some genes like SmPMRC1 and Musashi were highly up-regulated in mature ovaries. This may be due to the development of primary oocytes in these organs that are formed upon pairing. Previous studies have shown that steroid hormone signaling and translational regulation by Musashi are linked to oocyte maturation in model organisms (Hammes, 2004; Charlesworth et al., 2006; Arumugam et al., 2010; Parthasarathy et al., 2010). Other genes like Notch or both FGFRs were less affected by pairing, possibly because they are linked to stem cell control and stem cell fate (Dvorak et al., 2006; Gotoh, 2009; Liu et al., 2010; Waters and Reinke, 2011; Dalton, 2013; Koch et al., 2013).

The most remarkable results with respect to pairing-induced gene expression in gonads were those obtained for SmFz1 and Notch, emphasizing the advantages of tissue-specific analyses. Both genes seemed to be unaffected in their transcription by pairing when transcription rates of whole worms were compared. In contrast, transcription of both genes in testes and ovaries seemed to be strongly influenced by maturation processes. This demonstrates that “zooming-in” into specific tissues can unmask aspects of pairing-regulated gene expression, which otherwise remain undiscovered. Therefore, organ isolation combined with subsequent gene expression analyses lead to a more reliable identification of pairing-dependently expressed genes involved in processes linked to sexual reproduction. Since the latter is the prerequisite for initiating and maintaining egg production, this approach has the capacity to enlarge the repertoire of candidate genes envisaged for further evaluation with respect to new antischistosomals.

Because of its pairing-dependent expression in the gonads, SmFz1 was one of three genes characterized further. Sequence analyses confirmed homology to members of the frizzled family of seven transmembrane receptors for Wnt lipoglycoprotein-type growth factors (Schulte, 2010). Wnt/frizzled signaling is highly conserved throughout the animal kingdom and involved in a broad range of developmental processes during embryogenesis, organogenesis (Huang and Klein, 2004; Almuedo-Castillo et al., 2012) but also in carcinogenesis (Klaus and Birchmeier, 2008).

In *S. mansoni* at least four different frizzled receptors, SmFz1 - SmFz4, were identified by previous *in silico* analyses (Zamanian et al., 2011; Hahnel et al., unpublished), but up to now none of those genes have been investigated further. In the present study *in situ*-hybridization experiments localized SmFz1 transcripts in testes and ovaries of adult *S. mansoni* and confirmed the results of the gonad-specific qRT-PCRs. Because transcription was not detected in other tissues, we conclude that SmFz1 is gonad-specifically expressed. Since no transcripts of other frizzled receptors could be localized in the gonads by organ-specific RT-PCRs (data not shown), it seems likely that Fz-signaling in the gonads of *S. mansoni* is exclusively mediated by SmFz1.

Although frizzled receptors belong to the superfamily of GPCRs, typical canonical Frizzled-signaling pathways occur independently of heterotrimeric G proteins, but rather by interacting with the cytosolic adapter protein Disheveled (Dvl) (Gao and Chen, 2010). Since a characteristic Dvl-binding motif was found within the C-terminus of SmFz1, and SmDvl1 as well as SmDvl2 transcripts were identified in testes and ovaries by organ-specific RT-PCRs (data not shown), evidence exists that at least one SmFz1-dependent canonical frizzled pathway is expressed in the gonads of adult *S. mansoni*. Using an inhibitor (3289-8625), which specifically blocks Frizzled-Dvl interactions, we started to investigate the role of associated signaling pathways in adult *S.* mansoni *in vitro*. Adult worms treated with this inhibitor showed dramatic morphological alterations in the gonads accompanied by a massive destruction of cells in testes and ovaries. Because Frizzled-Dvl signaling is involved in controlling fundamental processes like organogenesis and gametogenesis in the gonads of diverse organisms (Schalburg et al., 2006; Golestaneh et al., 2009; Sirotkin, 2011) the observed phenotype might be due to inhibition of SmFz1-signaling in these tissues. Furthermore, worm vitality and morphology were affected, leading to the conclusion that further Frizzled-Dvl pathways were also expressed in other tissues of adult *S. mansoni* and additionally targeted by the inhibitor. Finally, effects were also detected in eggs laid by treated couples. As 3289-8625 prevented normal embryo development in such eggs, the obtained results provide first evidence for the involvement of Frizzled-Dvl signaling in early embryogenesis of *S. mansoni* as has been reported also for other organisms (Huang and Klein, 2004; van Amerongen and Nusse, 2009; Wansleeben and Meijlink, 2011; Almuedo-Castillo et al., 2012). Although an IC_50_ of 12.5 μM for 3289-8625 in human cell culture was reported (Grandy et al., 2009), the effects observed here on worm morphology and embryogenesis occurred at relatively high inhibitor concentrations. This discrepancy might be explained by structural differences of the inhibitor target sites of human and schistosome homologs. Even though the PDZ domains of SmDvl1 and SmDvl2 shared high similarity to those of human Dvl2 with sequence identities of 72.5 and 85%, respectively, we cannot exclude that key residues are missing in the schistosome homologs which are involved in inhibitor binding. In addition, the uptake of the compound by the parasite, its chemical stability in worm culture medium and tissue, as well as its distribution inside the worm, are potential factors influencing inhibitor activity.

As Frizzled receptors, RTKs of the FGFR family are highly conserved throughout the *Eumetazoa* (Itoh and Ornitz, 2004, 2011) and control a wide spectrum of cellular processes including cell division, differentiation, maintenance, migration and apoptosis (Powers et al., 2000). Therefore, they fulfill a crucial role in organogenesis during embryonic development of both, invertebrates and vertebrates, but are also involved in the regulation of physiological processes like tissue regeneration, homeostasis, and angiogenesis in the adult organism (Turner and Grose, 2010). Additionally, several studies have linked FGFR signaling to stem cell control in different model systems (Dvorak et al., 2006; Gotoh, 2009; Dalton, 2013). FGFRs are also expressed in planarian neoblasts. They represent pluripotent stem cells involved in somatic tissue regeneration of these free-living flatworms (Ogawa et al., 2002; Adell et al., 2010).

In schistosomes, two homologs of the FGFR-family exist, SmFGFR-A and SmFGR-B. Sequence analyses revealed that SmFGFR-A exhibits characteristic features of FGFRs including two extracellular IG-like domains sufficient for ligand binding and receptor dimerization (Eswarakumar et al., 2005). SmFGFR-B seems to represent a truncated form as the extracellular N-terminus lacks identifiable domains. However, GVBD-assays performed in this study showed that both FGFRs possessed enzymatic activity independent of their extracellular domains, which provides evidence for their biological activity. Interestingly, our data suggest that regulation of the SmFGFR-A kinase activity occurs in a non-FGFR like fashion. Normally, FGFRs share the common regulatory principle of most RTKs that depends on ligand-induced receptor dimerization followed by a trans-phosphorylation of the activation loops of both TK domains, which is sufficient for enzymatic activity (Krauss, 2008). In contrast to SmFGFR-B and other non-schistosome FGFRs, SmFGFR–A contains a constitutively active TK domain, which enabled the induction of GVBD in its wild type form. This can be explained by the unusual structure of SmFGFR–A, as the TK domain contains variations of the amino acid sequence within the activation loop. Instead of a positive charged amino acid residue found in common FGFRs, a negatively charged glutamate is present at position GYME^781^ of SmFGFR–A. Thus, charging of regulatory tyrosines within the activation loop via phosphorylation seems not to be necessary for activation.

A similar phenomenon has been reported for epidermal growth factor receptors (EGFRs), where enzymatic activity is triggered by conformational changes of both TK domains following receptor dimerization but independent of phosphorylation processes inside the activation loop (Bose and Zhang, 2009). Because SmFGFR-A shares no remarkable homology to EGFRs it remains unclear if the receptor is regulated by similar mechanisms or yet another unknown way. Although SmFGFR-A and SmFGFR-B represent members of the FGFR family, both differ from typical structures by alterations in their TK domains or their extracellular region, respectively. This leads to the conclusion that regulation of FGFR signaling in schistosomes may occur by as yet unknown mechanisms, which will be the focus of further studies.

A former study identified SmFGFR-A as a key molecule in the regulation and maintenance of proliferating somatic cells (PSCs) in adult *S. mansoni* (Collins et al., 2013). The SmFGFR–A expressing cells showed neoblast-like stem cell characteristics and may represent a source for tissue regeneration (Collins et al., 2013). They were found to be distributed throughout the parenchyma of adult males and females, and formed clusters near the intestine (Collins et al., 2013). Interestingly, we were able to detect transcripts of SmFGFR-B in the parenchyma by *in situ* hybridization, which might be a first hint that the second schistosome FGFR is also involved in PSC regulation. Regarding a potential role of FGFRs in reproduction of schistosomes, Collins et al. (2013) detected SmFGFR-A expression in the testes of male worms, which was confirmed by our experiments. Additionally, we were able to localize SmFGFR–A transcripts in the ovary of females. Furthermore, transcripts of SmFGFR-B were also detected in these tissues, which provide evidence that both receptors function in signal transduction processes in the schistosome gonads. By analogy to PSC regulation, we hypothesized that the mitotic activity of germinal stem cells, might also be regulated by FGFR signaling. Interestingly, while both FGFRs were co-localized in the gonads, our localization results indicate that only SmFGFR-B is expressed in the vitellarium, where it could be involved in the regulation of stem cell-like S1-vitellocytes (Kunz, 2001).

To investigate the role of FGFR-signaling in schistosome reproduction we performed functional analyses using the angiokinase inhibitor BIBF1120 (Hilberg et al., 2008; Roth et al., 2009). This compound (also named Vargatef) inhibits enzymatic activity of human VEGFRs, PDGFRs, and FGFRs by blocking the ATP-binding site of the TK domain. As no VEGFR and PDGFR homologs are encoded in the *S. mansoni* genome we assume that SmFGFR-A and SmFGFR-B may represent major targets of the inhibitor in this parasite. GVBD-assays confirmed that BIBF1120 was able to block enzymatic activity of both FGFRs in a concentration-dependent manner. Interestingly, SmFGFR-B was affected at lower concentrations, showing a higher sensitivity of this homolog to the inhibitor. According to our localization studies, the GVBD-data, and the known association of FGFR-A to PSCs, we hypothesize that BIBF1120 possesses the capacity to alter PSCs function in adult *S. mansoni* but also FGFR-expressing cells in the reproductive organs. Treatment of *S. mansoni* couples with BIBF1120 *in vitro* showed severe effects on worm vitality, morphology, and reproduction using concentrations of 5–10 μM. Most obviously, treated worms exhibited a drastically swollen intestinal tract. This may explain the decreased viability observed following treatment. Because FGFR-B expression was also detected in the gastrodermis it seems to be conclusive that the inhibitor had a direct effect on this tissue. Furthermore, it seems likely that these alterations were triggered by a targeting of FGFR-signaling in PSCs, since it has been suggested by Collins et al. (2013) that gastrodermis renewal depends on PSC activity. Besides this, inhibitor treatment also affected the gonads, which contained damaged cells. This confirms that FGFR-signaling plays an important role in these tissues as well. Although FGFR-B expression was localized in the vitellarium, CLSM analysis of treated worms (5–10 μM) showed no obvious morphological changes in this organ. Lower inhibitor concentrations (1 μM) led to less dramatic phenotypes on vitality and worm morphology. Nevertheless, egg production of treated couples was strongly affected. In males, testes morphology and sperm production were altered upon treatment. The anterior part of ovaries in treated females contained degenerated immature oocytes, not clearly distinguishable from each other. Both phenotypes could be explained by an effect of BIBF1120 on mitotically active cells in these organs.

To test the hypothesis that BIBF1120 affects germinal stem cells we co-cultured inhibitor-treated couples with EdU. In correspondence to the results obtained by Collins et al. (2013) we detected EdU^+^-cells widely distributed throughout the parenchyma, along the intestine as well as in the gonads and the vitellarium of adult *S. mansoni*. Targeting FGFR-signaling using BIBF1120, however, led to a remarkable decline of PSCs in the parenchyma of both genders. Beyond this we observed the disappearance of mitotically active cells in the testes and in the vitellarium, spermatogonia and S1-vitellocytes, respectively. In contrast to these findings, oogonia in the ovaries of mature females seemed to be less sensitive to inhibitor treatment. Nevertheless, the effects of BIBF1120 on gonadal cells and S1-vitellocytes provide an explanation for the inhibitor-induced decline in egg production.

Taking into account the limitations of inhibitor usage for gene characterization, the results obtained here provide first hints for an involvement of FGFR-signaling in the reproductive biology of adult *S. mansoni.* Furthermore, the data of the previous and the present studies suggest that FGFR-signaling should be assessed as a target for alternative strategies fighting schistosomiasis.

### Conflict of interest statement

The authors declare that the research was conducted in the absence of any commercial or financial relationships that could be construed as a potential conflict of interest.

## Supplementary material

Figure S1 **Sequence alignment of the activation loop of diverse FGFRs** Sequence alignment of the activation loop of the human FGFR 3 (hFGFR3) (AC: AAI53825.1), DjFGFR1 (AC: Q8MY86.1), and DjFGFR2 (AC: Q8MY85.1) from the planarian *Dugesia japonica* as well as SmFGFR-A and SmFGFR-B. The DFG motif (blue box) is part of the Mg^2+^ binding pocket of the FGFR TK domain and is conserved in all analyzed receptors. This amino acid sequence was changed to DNA by site-directed mutagenesis to create inactive kinase domains of SmFGFR-A and SmFGFR-B, serving as negative controls in the GVBD assays. The green box highlights the conserved regulatory motif YYKK^650^ of (h)FGFR3 and the corresponding motif YYRK, occurring in both planarian receptors and SmFGFR-B. In (h)FGFR3 phosphorylation of both of the regulatory tyrosine residues N-terminal of K^650^ unblocks the catalytic site of the enzyme. The K^650^E mutation mimics this phosphorylation by introducing a negative charge and leads to a constitutively active kinase. For this reason a constitutively active variant of the SmFGFR-B TK domain (SmFGFR-B_TK-active) was generated by changing YYRK^519^ to YYRE^519^. In contrast, SmFGFR-A has the motif GYME^781^, containing a negatively charged glutamate C-terminal to the presumptive regulatory tyrosine residue (underlined). Thus, SmFGFR-A possesses a constitutively active TK domain in its wild type form, which is sufficient to induce GVBD.
