# Supplementary material for: Gonad RNA-specific qRT-PCR analyses identify genes with potential functions in schistosome reproduction such as SmFz1 and SmFGFRs
Source: Front Genet. 2014 Jun 10;5:170. doi: 10.3389/fgene.2014.00170 (PMC4050651; doi:10.3389/fgene.2014.00170)
Supplement: Figure S1 — Sequence alignment of the activation loop of diverse FGFRs Sequence alignment of the activation loop of the human FGFR 3 (hFGFR3) (AC: AAI53825.1), DjFGFR1 (AC: Q8MY86.1), and DjFGFR2 (AC: Q8MY85.1) from the planarian Dugesia japonica as well as SmFGFR-A and SmFGFR-B. The DFG motif (blue box) is part of the Mg2+ binding pocket of the FGFR TK domain and is conserved in all analyzed receptors. This amino acid sequence was changed to DNA by site-directed mutagenesis to create inactive kinase domains of SmFGFR-A and SmFGFR-B, serving as negative controls in the GVBD assays. The green box highlights the conserved regulatory motif YYKK650 of (h)FGFR3 and the corresponding motif YYRK, occurring in both planarian receptors and SmFGFR-B. In (h)FGFR3 phosphorylation of both of the regulatory tyrosine residues N-terminal of K650 unblocks the catalytic site of the enzyme. The K650E mutation mimics this phosphorylation by introducing a negative charge and leads to a constitutively active kinase. For this reason a constitutively active variant of the SmFGFR-B TK domain (SmFGFR-B_TK-active) was generated by changing YYRK519 to YYRE519. In contrast, SmFGFR-A has the motif GYME781, containing a negatively charged glutamate C-terminal to the presumptive regulatory tyrosine residue (underlined). Thus, SmFGFR-A possesses a constitutively active TK domain in its wild type form, which is sufficient to induce GVBD. [file Presentation1.ZIP › Supp Table 1.DOCX]

**Supplementary Table 1: Primers used for qRT-PCR analyses**

| **Gene name** | **GeneID** | **5'Primer sequence (5'→3')** | **3'Primer sequence (5'→3')** |
| --- | --- | --- | --- |
| SmFGFR-A | Smp_175590 | TGCATGTACCCAAGAGGAATCA | GATATGGTGAATTGCCCAAACTG |
| SmFGFR-B | Smp_157300 | CACAGAAGGAGATGTGTCTGAA | TTCCCGTAAGGAGCATATTCCA |
| SmFz1 | Smp_118970/  Smp_173940 | TGCACCGTCTGGAGTAACTG | GACCAGAATTAGCGGGTACG |
| SmPMRC1 | Smp_093700 | CCTAAGGGAATGGGAACTACA | ATGGTTGGTTCCAACGTCATCT |
| Notch | Smp_050520 | TGTAATCGTGGCAGCTATGG | TAGGGCCACACGGAGTTAAT |
| Musashi | Smp_157750 | ATCGTCGTAGCCTCAAGGT | GCAAAGCCTCTATGACGTG |
| Actin | Smp_161930 | GGAAGTTCAAGCCCTTGTTG | TCATCACCGACGTAGCTGTC |
